# A Simple Biorefinery Concept to Produce 2G-Lactic Acid from Sugar Beet Pulp (SBP): A High-Value Target Approach to Valorize a Waste Stream

**DOI:** 10.3390/molecules25092113

**Published:** 2020-04-30

**Authors:** Regiane Alves de Oliveira, Roland Schneider, Betânia Hoss Lunelli, Carlos Eduardo Vaz Rossell, Rubens Maciel Filho, Joachim Venus

**Affiliations:** 1 Laboratory of Optimization, Design and Advanced Process Control, School of Chemical Engineering, University of Campinas (Unicamp), Avenida Albert Einstein 500, Campinas 13083-852, Brazil; maciel@feq.unicamp.br; 2 Department of Bioengineering, Leibniz Institute for Agricultural Engineering and Bioeconomy e.V. (ATB), Max-Eyth-Allee 100, 14469 Potsdam, Germany; rschneider@atb-potsdam.de; 3 Pontifícia Universidade Católica de Campinas (PUC-Campinas), Centro de Ciências Exatas, Ambientais e de Tecnologias, Faculdade de Química, Rua Professor Doutor Euryclides de Jesus Zerbini 1516, Campinas 13087-571, Brazil; belunelli@gmail.com; 4 Interdisciplinary Center of Energy Planning (NIPE), University of Campinas (Unicamp), Rua Cora Coralina 330, Campinas 13083-896, Brazil; cevazrossell@gmail.com

**Keywords:** lactic acid, sugar beet pulp, biorefinery, second-generation, bipolar membrane electrodialysis

## Abstract

Lactic acid is a high-value molecule with a vast number of applications. Its production in the biorefineries model is a possibility for this sector to aggregate value to its production chain. Thus, this investigation presents a biorefinery model based on the traditional sugar beet industry proposing an approach to produce lactic acid from a waste stream. Sugar beet is used to produce sugar and ethanol, and the remaining pulp is sent to animal feed. Using *Bacillus coagulans* in a continuous fermentation, 2781.01 g of lactic acid was produced from 3916.91 g of sugars from hydrolyzed sugar beet pulp, with a maximum productivity of 18.06 g L^−1^h^−1^. Without interfering in the sugar production, ethanol, or lactic acid, it is also possible to produce pectin and phenolic compounds in the biorefinery. The lactic acid produced was purified by a bipolar membrane electrodialysis and the recovery reached 788.80 g/L with 98% *w*/*w* purity.

## 1. Introduction

Sugar beet provides 16% of the sugar produced worldwide [1]. In 2017, more than 300 Mt of sugar beet were produced in the world. Currently, Europe is responsible for around 70% of the total production. France and Germany together produced more than 68 Mt of sugar beet in 2017, which corresponded to more than 22% of the world production [2]. In 2017, the European regulation for the quotes and minimum sugar prices was abolished, which dramatically increased the competition and caused a decline in the prices [3]. The European Union, to the detriment of sugar producers, also has health-education campaigns aimed to reduce sugar consumption [3,4]. Considering this scenario, it is expected that sugar producers will look for new business models to diversify the sugar beet industry revenue [4].

In the traditional process, sucrose is extracted from sugar beet using hot water. The juice is then purified, concentrated, and crystallized. The remaining pulp is sent to animal feed or biogas production. The molasses is generally used for ethanol production, in animal feed, or as a medium for yeast biomass production [4]. Nowadays, with increasing biotechnological advancements, this process may be amplified to reach products that are more advantageous and sustainable. Considering this opportunity, this work proposes a new sugar beet biorefinery. 

**Figure 1:**
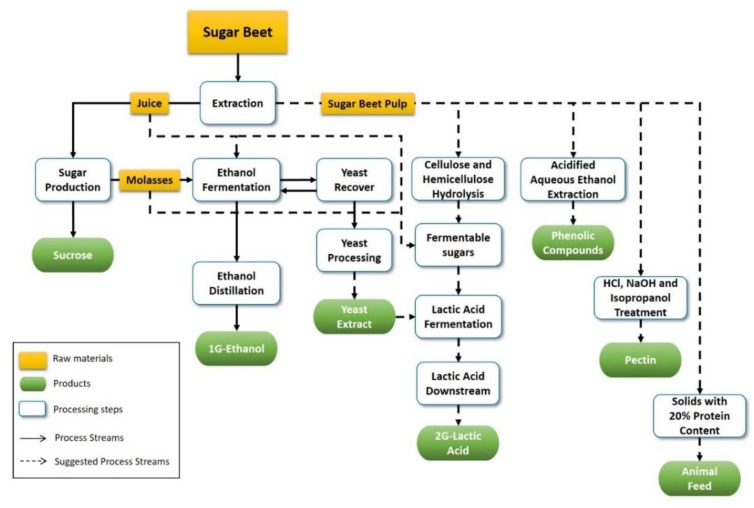
Sugar beet biorefinery: production of sucrose, ethanol, yeast products/yeast extract, lactic acid, phenolic compounds, pectin, and animal feed.

A biorefinery is an installation similar to an oil refinery that uses a conversion of biomass to produce biofuels, energy, and chemicals [5]. Considering the sugar beet as the main raw material, the proposed biorefinery may produce sucrose, ethanol, yeast products/yeast extract, lactic acid, phenolic compounds, pectin, and animal feed, as shown in Figure 1.

The production of sucrose and ethanol is a long-time known process for the sugar beet industry. However, the concept of biorefinery to aggregate value to its residues has just recently come into highlight [4,6]. The main residue from this industry, with a large potential to be used in the biorefinery, is the pulp remaining after the juice removal. Sugar beet pulp (SBP) is normally used as an animal feed [7,8,9,10]. Recently, it has been highlighted due to its relatively high amounts of hemicellulose and pectin to produce ethanol, methane, biogas, and acetone–butanol–ethanol [8,9,11,12,13,14,15,16,17]. However, bio-digestion is the only approach that is being currently used [4].

The current study presents a new possibility to produce second-generation (2G) lactic acid from SBP. Lactic is an organic acid with widespread applications mainly in pharmaceutical, cosmetic, chemical, as well as food industries [18,19]. In 2016, the global market required 1220.0 kt of lactic acid, and the demand is expected to grow 16.2% per year until 2025 [20]. Due to this, the production of lactic acid from SBP can mark a turning point in the restructuring of the sugar beet industry. The proposed model has a flexible design that can be compared to the sugarcane biorefineries. In this context, it is possible to shift the production chain from one product to another to meet the market demand for products with a higher benefit. Bearing all this in mind, this study proposes a new approach to the sugar beet industry, amplifying the possibilities of final products with a higher added value.

## 2. Results and Discussion

### 2.1. Lactic Acid Fermentation

*B. coagulans* is a homofermentative microorganism that efficiently converts the hydrolyzed SBP into lactic acid. The main results from the carried-out fermentations are presented below.

Two batch fermentations (named B1 and B2) were performed simultaneously in identical bioreactors, to evaluate the need for yeast extract addition. In batch B1, the use of yeast extract resulted in a total lactic acid production of 29.90 g, with a yield of 0.83 *g*/*g*, an average productivity of 1.25 g L^−1^h^−1^, and a maximum productivity of 12.48 g L^−1^h^−1^. Yeast extract is used as an effective source of nutrients for high lactic acid production, especially due to the presence of vitamin B and amino acids used for cell growth [19]. However, yeast extract addition may also result in a higher cost for lactic acid production [21]. Bearing this in mind, and considering SBP has a considerable protein content, protease was added to batch B2 during the enzymatic hydrolysis to release the amino acids present in the SBP, instead of adding yeast extract in the fermentation broth. The SBP used in this study contained ≈ 9% of proteins on a dry basis, which can be highly advantageous for lactic acid production since lactic acid bacteria have a high need for amino acids in the culture media [21]. Using this approach, the lactic acid production reached 29.14 g, with a yield of 0.73 g/g, average productivity of 1.21 g L^−1^h^−1^, and maximum productivity of 14.67 g L^−1^h^−1^. Comparing batches B1 and B2 (Table 1), it is noticeable that yeast extract presence improved the yield. However, lactic acid concentration and average productivities were similar using either protease or yeast extract. However, considering the maximum productivity, B2 batch presented better results, with a lesser amount of remaining sugar after 24 h. For those reasons, it was decided to use protease in the enzymatic hydrolysis, and to operate the fermentations in the absence of yeast extract.

**Figure 2:**
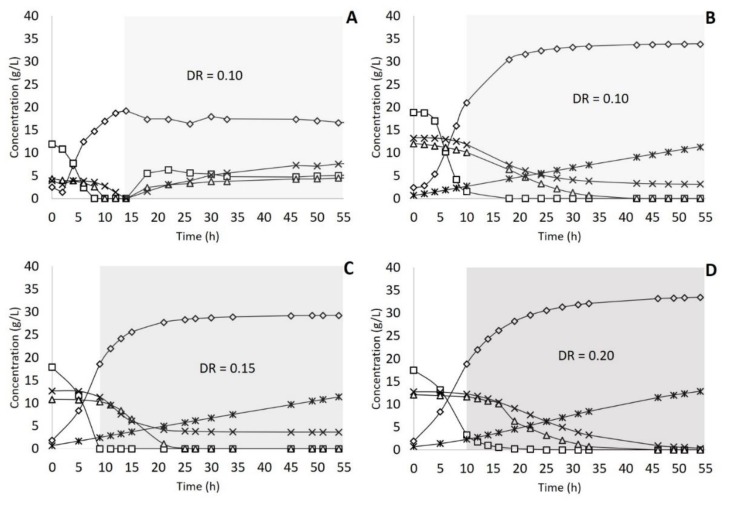
Continuous fermentation of sugar beet pulp (SBP) hydrolysate. (**A**) Continuous process (C1) without cell retention and dilution rate = 0.10 h^−1^; (**B**) Continuous process (C2) with cell retention and dilution rate = 0.10 h^−1^; (**C**) Continuous process (C3) with cell retention and dilution rate = 0.15 h^−1^; (**D**) Continuous process (C4) with cell retention and dilution rate = 0.20 h^−1^; (◊) Lactic acid; (□) Glucose; (∆) Xylose; (×) Arabinose; (∗) Biomass; DR = dilution rate.

In the next step, continuous fermentations were used to evaluate the impact of a cell retention membrane on lactic acid production. In the first case, C1, the continuous system had no cell retention membrane, and the dilution rate was fixed at 0.10 h^−1^. Figure 2A shows the lactic acid production and consumption of sugars over the process. In this case, glucose was depleted after 8 h of fermentation, and the feed started after 14 h. The maximum lactic acid concentration reached 19.26 g/L at 14 h of fermentation. After this time, the lactic acid concentration started a light decrease, as shown in Figure 2A. After 54 h, it was possible to produce 296.19 g of lactic acid from 1158.58 g of sugars. The average productivity and yield were 0.27 g L^−1^h^−1^ and 0.38 *g*/*g* (Table 1), and 379.13 g of sugars remained unconsumed, which represents more than 30% of the total sugars available.

Based on these results, an identical fermentation (C2) was performed using an annexed cell retention system. The dilution rate was kept the same as experiment C1 (0.10 h^−1^), and the feed started after 10 h when the glucose was almost depleted (around 2 g/L). The maximum lactic acid concentration reached 34.79 g/L after 33 h of fermentation (Figure 2B). In this case, the global yield was 0.76 *g*/*g*, and the average productivity was 0.56 g L^−1^h^−1^. After 54 h of the process, 450.98 g of lactic acid was produced (Table 1). Arabinose was not totally consumed; 145.83 g remained after 54 h of fermentation.

Comparing the experiments C1 and C2, it is possible to state that the cell retention system positively impacted the lactic acid production from hydrolyzed SBP. The maximum lactic acid concentration increased by more than 44% in fermentation C2. Besides, all the analyzed parameters improved, such as yield, average and maximum productivities, remaining sugars, and total lactic acid produced. An explanation for those differences in the results is the fact that, in the absence of a cell retention system, the biomass produced to convert the sugars into lactic acid was constantly being washed out of the system. Thus, it was determined to proceed with the tests using the cell retention system.

Fermentations C2, C3, C4, and C5 were performed for 54 h with different dilution rates (Table 1): 0.10 h^−1^, 0.15 h^−1^, 0.20 h^−1^, and 0.30 h^−1^. C2, C3, and C4 presented similar results for yield, average productivity, and µP max. As expected, increasing the dilution rate increased the lactic acid production, maximum productivity (from 11.60 to 16.21 g L^−1^h^−1^), and also the remaining sugars as follows (Table 1): 14% for C2, and 19% for C3 and C4, all mainly composed of arabinose. Figure 2B–D show the concentrations of the main components over 54 h of each process, namely C2, C3, and C4, respectively. However, in the C5 case, the increase of dilution rate to 0.30 h^−1^ resulted in more than 33% of non-consumed sugars and a considerable decrease in average and maximum productivities (Table 1).

**Figure 3:**
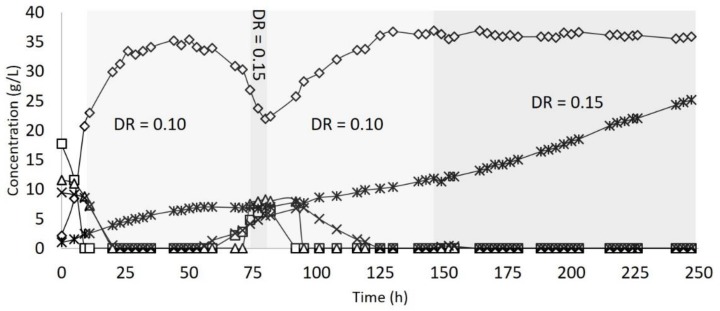
Continuous fermentation of SBP hydrolysate with cell retention and dilution rate (DR) variation. (◊) Lactic acid; (□) Glucose; (∆) Xylose; (×) Arabinose; (∗) Biomass; DR = dilution rate.

Finally, the latest test was performed based on the previous results in a continuous mode of fermentation with the cell retention system for 250 h. In this case, the maximum lactic acid concentration was 36.92 g/L. The feed started after 9 h of the process (initial glucose concentration depleted) with a dilution rate of 0.10 h^−1^, which was kept constant until 71 h when the cell concentration reached approximately 6.8 g/L on a dry basis. At this point, the dilution rate was increased to 0.15 h^−1^ for 8 h, resulting in a significant decrease in the lactic acid production to 21.96 g/L and without an improvement in biomass production. Based on that, the dilution rate was decreased to 0.10 h^−1^, leading to an increase in the lactic acid concentration and the biomass amount. At 146 h, the biomass had almost doubled, reaching 12.0 g/L on a dry basis. Once more, the dilution rate was increased to 0.15 h^−1^, and with this cell concentration, the process was stable for more 104 h (Figure 3). The process was stopped after 250 h running without further complications, and the flow on the membrane used for cell retention was not blocked. As an overall result, 2781.01 g of lactic were produced from 3916.91 g of sugars, meaning a global yield of 0.71 *g*/*g* and no remaining sugars. The maximum productivity reached 18.06 g L^−1^h^−1^, and the µ maximum was 1.45, both the highest parameters obtained in the tests (Table 1).

This latest fermentation showed that SBP may be an excellent substrate to be employed as a substrate for lactic acid production, since its composition suited the needs of *B. coagulans* without any extra supplementation of nutrients, even for a long time with a running process. Additionally, the process temperature settled at 52 °C helped to avoid further contamination for everyday strains, making it possible to run the process for longer times without by-product production.

Considering the recent literature, López-Gómez et al. [22] produced 61.1 g/L of l-lactic acid from hydrolyzed municipal solid waste on a batch mode, noticing a high positive impact of yeast extract addition on the media. Balakrishnan et al. [23] produced 45.08 g/L of d-lactic acid from hydrolyzed millet bran residue and hydrolyzed casein, with a productivity of 0.45 g L^−1^h^−1^. Tian et al. [24] produced 114.6 g/L of lactic acid from purified sweet sorghum juice and soybean hydrolysate with a productivity of 2.61 g L^−1^h^−1^ on a simple batch mode. Using simultaneous saccharification and fermentation of lignocellulosic corncob residue, Jiang et al. [25] produced 79.1 g/L of l-lactic acid with a yield of 0.76 *g*/*g* cellulose in fed-batch mode. Solid carob waste from the Lebanese industry was also suggested by Bahry et al. [26], who studied *Lactobacillus rhamnosus* immobilization in alginates beads for lactic acid production, reaching a productivity of 1.22 g L^−1^h^−1^. d-lactic acid production from orange peel waste hydrolysates was shown by de la Torre et al. [27], using cell adaptation, to reach 6.72 g L^−1^h^−1^ of lactic acid productivity. Cell adaptation was also used as a strategy by Aulitto et al. [28] to produce lactic acid from simultaneous saccharification and lactic acid fermentation of steam exploded wheat straw, reaching lower process water requirements and a more cost-effective seed cultivation with physiological pre-adaptation of the production strain. All these results show broad possibilities for the sustainable production of lactic acid, using different substrates, according to their availability in specific regions. For SBP it is no different; regions with the availability of this substrate may include lactic acid production in their already installed facilities, contributing to a more sustainable and profitable economy.

Sugar beet pulp has gained attention due to its high amount of hemicellulose and pectin, moderate cellulose, and low lignin amounts [9]. Some processes for its utilization have been suggested, including the production of biogas [11,12], ethanol [8,12,13,14,15], methane [16], and acetone–butanol–ethanol [17]. More recent studies amplify the possibilities of more valuable products, such as succinic acid [6], pectin [6,29,30,31], and phenolic compounds [6,32]. Following this approach, a biorefinery was designed to produce lactic from sugar beet pulp, addressing value to a residue that is currently being mainly sent to animal feed.

### 2.2. Biorefinery Proposal Process Design

The biorefinery concept can be applied to lactic acid production from SBP, as it has been done for sugar and ethanol production. One of the advantages of using a byproduct of sugar production for lactic acid production is that its industrial plant can be easily integrated into a mill following the biorefinery concept. This may be also interesting for the economical point of view, since it provides a larger range of business opportunities. 

A simplified mass balance of an integrated first (1G) and 2G sugar beet proposed biorefinery was performed in Microsoft Excel based on literature data [6,33,34,35]. Due to the lack of information, the energy balances were not carried out. The plant processing capacity (dry t of sugar beets) was estimated to produce annually 20,000 t of lactic acid from sugar beet pulp. In addition to lactic acid, the proposed biorefinery configuration also produces sugar, ethanol, pectin, phenolic compounds, and animal feed with 20% protein. The overall industrial plant consists of 6 process units for the 1G plant: (i) slice and extraction, (ii) clarification, (iii) evaporation, (iv) sugar crystallization, (v) ethanol fermentation, and (vi) ethanol purification; and 5 process units for the 2G plant: (vii) free sugar extraction, (viii) acid/enzymatic pretreatment, (ix) centrifugation, (x) lactic acid fermentation, and (xi) lactic acid downstream.

**Figure 4:**
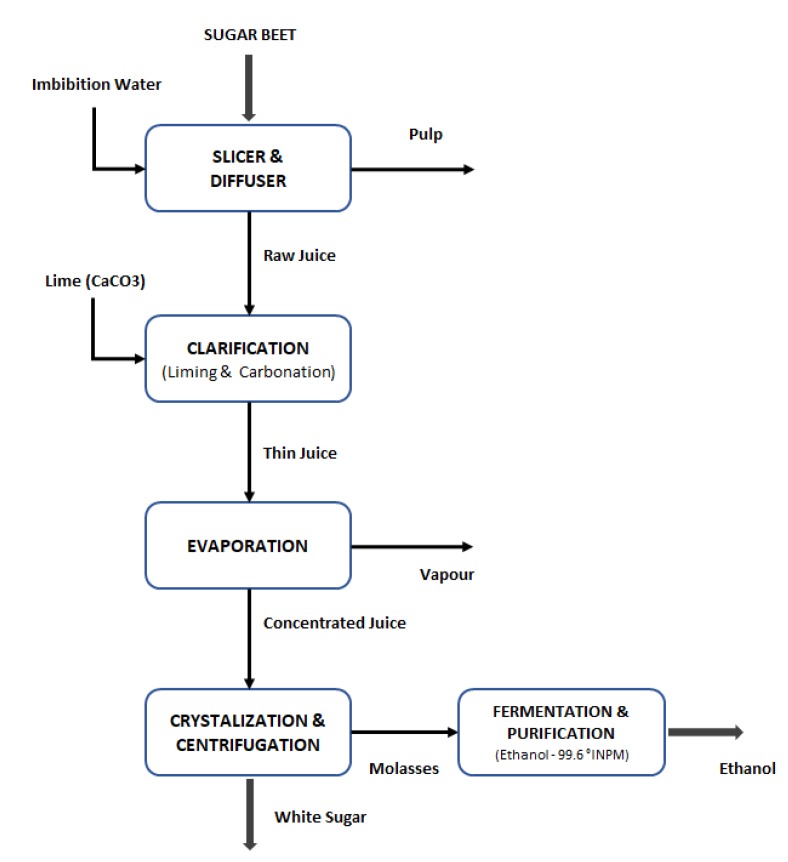
Traditional sugar beet processing to produce ethanol and white sugar.

All 1G products and inputs were gathered from Dorst, Krajnc et al., Lorenz, Marzo et al., and Zeist et al. [33,34,35,36,37]. The recovery efficiency of 98% was assumed in the sugar extraction unit. From this unit, a raw juice with a 14 Brix degree was obtained. The juice was concentrated in an evaporation system to increase the Brix degree to 65. The ethanol fermentation yield was fixed in 0.46 as reported in the literature [38], and a purification (distillation and dehydration) efficiency of 98% was assumed [38] to produce anhydrous ethanol with 99.2° GL (99.6%). Figure 4 shows the traditional sugar beet process to produce white sugar and ethanol. In this case, sugar is produced from sugar beet juice. Molasses is used for the production of ethanol. SBP, which is obtained after the juice extraction, is then diverted to the other products.

For this study, a medium-sized plant with a milling capacity of 340 days of operation was adopted. This plant has the potential to produce around 315,000 t of sugar and 13 million L of anhydride ethanol per year, considering 8 t molasses produced per hour. From this operation, 405,000 t of SBP is produced, to be designated to lactic acid production and other high-value products.

Finally, considering a goal of a minimum of 20,000 t of lactic acid production per year with 85% purity, an integrated process for a biorefinery was designed considering the experimental data from C2, C3, and C4. In this case, the industrial plant has the capacity of processing an average of 110 t of SBP per year, resulting in average production of 8600 t/year of phenolic compounds, 33,300 t/year of pectin, and 23,000 t/year of animal feed with 20% of protein content, as well as 20,000 t of lactic acid. The produced lactic acid is then recovered employing a liquid−liquid extraction process and a stripping separation, as presented by Udachan and Sahoo [39]. The recoveries and yields presented by Alexandri et al. [6] were used in the 2G process mass balances. Different conversions were considered for the lactic acid fermentation process to cover all the experiments performed (Table 2). In the downstream process (liquid–liquid extraction and stripping), a recovery of 85% was assumed [40].

**Figure 5:**
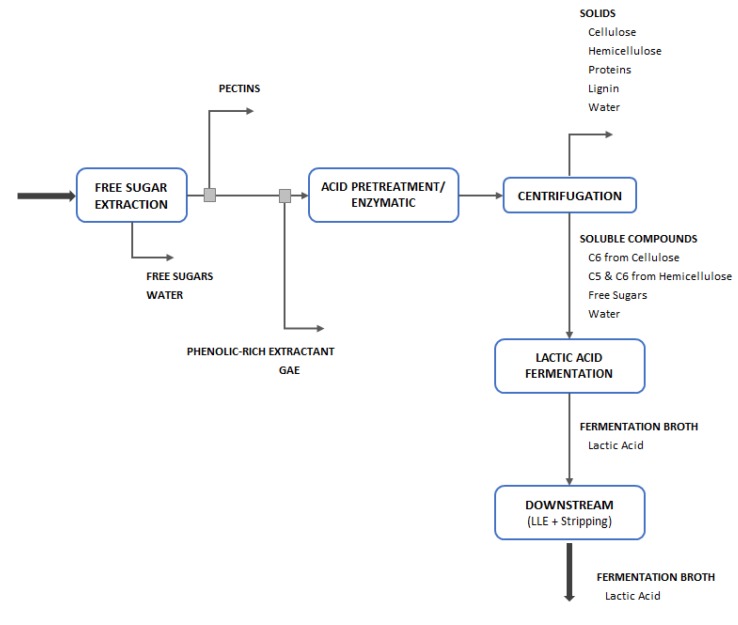
Integrated process diagram for lactic acid, pectin, phenolic compounds, and animal feed production.

Finally, considering the C6 fermentation data, Figure 5 presents an example of the integrated process diagram for lactic acid, pectin, phenolic compounds, and animal feed production from SBP. In this case, over 340 days of operation per year, it was necessary to process 97,000 t/year of SBP to produce 20,000 t/year of lactic acid, 7600 t/year of phenolic compounds, 29,200 t/year of pectin, and 20,000 t/year of animal feed with 20% of protein content.

### 2.3. Alternative Lactic Acid Downstream

The lactic acid downstream method proposed in this investigation is based on a bipolar membrane electrodialysis. Two fermentation broths were submitted to this process: C2, C3, and C4 mixed broths (to reach a working volume enough to the downstream process), and C6 fermentation broth.

C2, C3, and C4 mixed broths resulted in 62.90 L of liquid, with 29.84 g/L of lactic acid, 9.88 g/L of residual sugars, a considerable amount of SO_4_^2−^ resulting from the SBP pretreatment, and a considerable amount of Na+ from the lactic acid neutralization during the fermentation process (Table 3). The microfiltration to remove solids and microbial cells had already been performed with the cell retention system in the fermentation step. After the nanofiltration step, approximately 87% w/w of the lactic acid was recovered in the permeate stream. Next, this stream was submitted to a decolorization and a softening process for the removal of colorants, magnesium, and calcium salts. A monopolar electrodialysis was then carried out for the removal of residual sugars, resulting in a concentrated stream with 89.86 g/L of lactic acid. The bipolar electrodialysis generated three streams, with the lactic concentrated mainly in the acid one (110.30 g/L) and loss of less than 1% *w*/*w* in the salt stream. One more decolorization step was performed before the anion and cation exchange resins were used to remove the ions. The last step was a vacuum distillation process performed to concentrate the lactic acid. As result, it generated 1.10 L of concentrate with a lactic acid concentration of 788.80 g/L, free of residual sugars, and with a final lactic acid purity of 98% *w*/*w*. The main impurities were acetic acid (13.15 g/L) and Na^+^ (6.97 g/L). The overall process yield was 46.23% *w*/*w*, higher than the 23% found by Pleissner et al. [41] and the 38% found by Neu et al. [42] using similar methodologies. 

The C6 fermentation broth (88.00 L) was composed of 31.66 g/L of lactic acid, 1808.00 mg/L of SO_4_^2−^, and 12,819.43 mg/L of Na^+^ (Table 3). In this case, all sugars were consumed during the fermentation process. Table 3 shows that, in this case, some extra steps were performed. Besides the nine steps from the previous case, two more anion exchangers steps, one more cation exchanger step, and one more vacuum distillation step were added in an attempt to reduce the cation and anion impurities in the final stream. As a result, 1.35 L of concentrate was generated with a lactic acid concentration of 819.70 g/L and a final lactic acid purity of 95% *w*/*w*. The main impurities were acetic acid (40.00 g/L) and Na^+^ (8.98 g/L). The overall process yield was 39.72% *w*/*w*. As was expected, the increase in the number of steps led to a decrease in the process yield when compared to the previous one.

Using defined and semi-defined fermentation broth, Pleissner et al. [43] achieved a yield of more than 90% of lactic acid using electrodialysis. Differently, when more complex substrates were used in the fermentation process, a similar value was never found. To the best of our knowledge, the yield of 46.23% *w*/*w* presented in this work is the highest achieved using electrodialysis for the separation and purification of 2G-lactic acid produced from SBP. In general terms, membrane downstream processes are efficient for a separation between lactic acid and fermentation sugars because of their significant differences in molecular weight. Additionally, they are environmentally friendly and easy to scale up. For those reasons, bipolar membrane electrodialysis has attracted attention for organic acid separation and purification [44]. An important advantage of this method is the possibility of a base recovery without the addition of inorganic acids [42,44,45,46].

The use of electrodialysis for lactic acid separation and purification also has the advantage of recycling inputs that are necessary for previous steps, especially NaOH. The recovery and reuse of Na^+^ was already demonstrated by Pleissner et al. [43]. The authors showed that the regenerated NaOH solution did not jeopardize the fermentation.

The optimization of each step of this process could lead to higher product recovery yields and a decrease in the impurities. However, the average purity of 96.5% *w*/*w* lactic acid reached in this study was already more than the commercial lactic acid sold as a solution of 88% [47]. Furthermore, recirculating the retentate from nanofiltration, the base and salt streams from bipolar electrodialysis, and the diluate from monopolar electrodialysis may also reduce the lactic acid losses. Additionally, Wee et al. [48] proposed using the “waste” streams from electrodialysis directly back in the fermentation media, increasing the overall yield of the process. This process also offers the advantage of the inputs being recovered and reused [43], reducing the number of waste streams for the lactic acid production process.

The lactic acid downstream process is a fundamental step in the lactic acid production chain. Many applications require a final lactic acid of high purity and, together with this issue, comes the environmental concerns regarding solvent use and waste production [44]. According to Oliveira et al. [44], even though lactic acid downstream is commonly explored in the literature, 2G-lactic acid downstream has not been broadly explored. In previous works [49,50], our group demonstrated that processes that are well explored for other areas and compounds present different challenges when it comes to the downstream of 1G and 2G-lactic acid. As an example, hybrid short path evaporation presents very different results when it comes to the separation of lactic acid produced from molasses and the one produced from hydrolysates [49]. This means the interaction of the lactic acid molecule with the other media components has a great impact on the downstream process to be chosen when it comes to more sustainable lactic acid production [49].

## 3. Materials and Methods

### 3.1. Microorganism—Bacillus coagulans A166

*Bacillus coagulans* A166 strain (Leibniz-Institut für Agrartechnik und Bioökonomie e.V. (ATB), Leibniz, Germany) used in the fermentation was isolated from hemp leaves. The microorganism was stored in glycerol 30% *v*/*v* in a freezer at −80 °C.

The inoculum was grown in 250 mL sterile Erlenmeyer flasks with 60 mL MRS [51] broth (Merck, Darmstadt, Germany) and 0.67 g Everzit Dol (Evers, Hopsten, Germany) dolomite as the buffer. The initial pH was adjusted to 6.0 ± 0.1. Cultivation was carried out at 52 °C under stirring at 100 rpm for 16 h in an orbital shaker.

### 3.2. Sugar Beet Pulp

Pfeifer and Langen GmbH and Co. KG (Cologne, Germany) provided sugar beet pulp (SBP) in the form of dry pellets. The material was composed of 31.6% hemicellulose, 21.5% cellulose, 9.7% proteins, 8.7% free sugars, 8.5% ash, 2.1% lignin, 2.1% fat, and approximately 15% non-identified material.

### 3.3. Sugar Beet Pulp Hydrolysis

Based on a previous study from Alexandri et al. [6], 10% *w*/*w* of SBP was pre-treated at 121 °C for 30 min in the presence of H_2_SO_4_ 0.5% (*v*/*v*). Afterward, the pH was adjusted manually to 5.0 ± 0.1 by adding NaOH 20% (*w*/*w*). The pre-treated SBP was transferred to a 2 L BIOSTAT bioreactor (Sartorius AG, Göttingen, Germany) in a lab-scale, and to a 50 L bioreactor (B-Braun Biotech, Germany) in a pilot-scale, both assembled to 50 °C, 150 rpm and pH of 5.0 ± 0.1. In these conditions, pre-treated SBP was enzymatically hydrolyzed for 24 h using the following:

- 0.3 mL of Accellerase 1500/g of cellulose
- 0.114 mL of Pectinase L40/L of pre-treated SBP
- 0.429 mL of Protease Fermgen/L of pre-treated SBP

The average sugar concentration in the hydrolyzed SBP was 16.48 ± 2.77 g/L of glucose; 9.62 ± 3.38 g/L of xylose; and 9.98 ± 3.69 g/L of arabinose.

### 3.4. Fermentation

#### 3.4.1. Batch Fermentation

Fermentations using SBP hydrolysate were carried out in duplicate at 52 °C, 400 rpm, and pH 6. The bioreactor used was a 2 L BIOSTAT bioreactor (Sartorius AG) with 1 L of working volume. Regulation of pH was carried out by an automatic injection of 20% (*w*/*w*) NaOH. Samples were taken regularly for the analysis of sugar (glucose, xylose, and arabinose), lactic acid, and other organic acid concentrations, as well as for dry cell weight. After cell inactivation for 20 min at 90 °C, samples were stored at −20 °C.

#### 3.4.2. Continuous Fermentation

**Figure 6:**
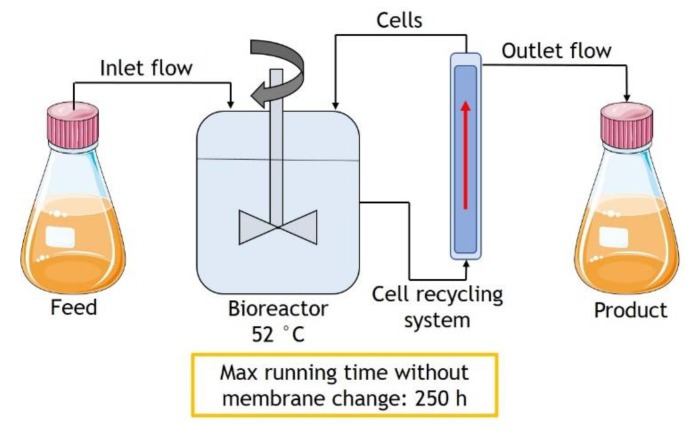
Schematic representation of the continuous fermentation system with cell retention.

Continuous fermentations were carried out in a sterile 5 L BIOSTAT bioreactor (Sartorius AG). The initial working volume was 3 L. Sterile SBP hydrolysate was used as a carbon and nutrient source for the microorganism. The pH was adjusted to 6 by injection of 20% (*w*/*w*) NaOH. Temperature and stir were fixed at 52 °C and 300 rpm. After the consumption of all the glucose present in the hydrolysate, the continuous process was started. The feed composition was sterile SBP. Different dilution rates were evaluated: 0.10; 0.15; 0.20; 0.30 h^−1^. The level was fixed at 3 L. The cell retention module system was equipped with hollow-fiber filters UMP-1147 (Pall Corporation, Port Washington, NY, USA), with a pore size of 0.2 µm. A schematic representation of the system is shown in Figure 6. The broth containing the lactic acid produced was stored in a cold room at 8° C to be used in the downstream process. Samples were taken regularly for the analysis of sugar (glucose, xylose, and arabinose) and lactic acid concentrations, as well as for dry cell weight. After cell inactivation for 20 min at 90 °C, samples were stored at −20 °C.

### 3.5. Down-Stream Processing—Electrodialysis

The downstream process was performed in the following sequenced steps:

- Decolorization was performed using PUROLITE MN-502 (Purolite, Ratingen, Germany)
- Softening was carried out using PUROLITE S950 acid chelating resin (Purolite).
- Mono- and bi-polar electrodialysis were carried out with 11 cation exchange membranes Type II and 10 anion exchange membranes Type II (both from Fujifilm, Tilburg, Netherlands)
- Anion and cation exchange chromatographies were performed using a weak anion exchange resin RELITE EXA 133 and a strong cation exchange resin RELITE EXC 08 (both from Resindion S. R. L., Binasco, Italy)
- Concentration was done by vacuum distillation plant (Büchi Labortechnik, Essen, Germany)

Details about the operational procedures can be found in a previous work by Neu et al. [42].

### 3.6. Analytics

The dry matter of SBP was determined by weighing a certain amount and then drying it at 105 °C until constant weight. The content of cellulose, hemicellulose, and lignin of SBP was determined using an ANKOM2000 fiber analyzer [52]. The SBP protein content was calculated following the procedure of total Kjeldahl-nitrogen according to the DIN-EN-25663 standard method [53]. 

For dry cell weight, the sample was centrifuged to separate the cells. The cell pellets were washed and placed in a pre-weighed cup, which was dried in an incubator at 80 °C for 24 h. After cooling to room temperature, the cup was weighed again, and cell dry weight was calculated. This procedure was carried out in duplicate. 

Organic acids and sugars were analyzed using HPLC (Dionex, Sunnyvale, CA, USA); 10 μL of sample volume (auto-sampler: Analytical WPS-3000TSL) was added on a Eurokat H column (300 mm × 8 mm × 10 μm, Knauer, Germany) and eluted isocratically with 0.8 mL/min of 0.01 M H_2_SO_4_. Detection was carried out by a refractive index detector (RI-101, Shodex, Tokyo, Japan). The total run time was 17 min. Analysis of HMF and furfural were carried out using a column C18 Eurosphere II (KNAUER, Berlin, Germany). The mobile phase was acetonitrile in water (1:8) with 1% *v*/*v* of acetic acid. The flow was 1.0 mL/min at 23 °C. Detection was carried out by a UV/VIS-Detektor at 280nm. The isomeric purity was analyzed using HPLC (KNAUER) coupled with a Chiralpak^®^MA(+) column (Daicel, Tokyo, Japan, 50 mm × 4.6 mm × 3 μm) and an ultraviolet detector. The mobile phase was 2 mM CuSO_4_, and the flow rate was 0.8 mL/min.

## 4. Conclusions

SBP is a promising substrate for lactic acid production, approaching a sustainable biorefinery production mode. As a substrate to second-generation lactic acid production, it can reconfigure the sugar beet industry, inserting this crop in the modern biorefinery concept of product valorization and sustainability. This biorefinery can become a supplier for different industrial sectors, including traditional food, energy, and feed, to a more complete chain, encompassing lactic acid, phenolic compounds, and pectin production. In addition, bipolar membrane electrodialysis can be adjusted to perfectly fit the sustainable needs of the lactic acid industry, generating zero waste towards a more sustainable process. 

## Figures and Tables

**Table 1:** Main results obtained from the different fermentation processes with *Bacillus coagulans* A166. The values were obtained through the mass balance calculations.

	Fermentation Mode	Time	Total Sugars*	Non-Consumed Sugars*	Lactic Acid Produced	Yield	Average Productivity	Max Productivity	µP Max
		h	g	g	g	*g*/*g*	g L^−1^h^−1^	g L^−1^h^−1^	h^−1^
B1	Batch, yeast extract	24	41.05	5.03	29.90	0.83	1.25	12.48	-
B2	Batch, protease	24	41.39	1.47	29.14	0.73	1.21	14.67	-
C1	Continuous, protease, no cell retention, DR = 0.10	54	1158.58	379.13	296.19	0.38	0.27	9.63	-
C2	Continuous, protease, cell retention, DR = 0.10	54	739.23	145.83	450.98	0.76	0.56	11.60	1.09
C3	Continuous, protease, cell retention, DR = 0.15	54	1322.45	254.10	779.90	0.73	0.57	14.25	1.03
C4	Continuous, protease, cell retention, DR = 0.20	54	1406.58	268.51	887.69	0.78	0.60	16.21	1.05
C5	Continuous, protease, cell retention, DR = 0.30	54	2116.42	707.21	972.36	0.69	0.38	9.69	-
C6	Continuous, protease, cell retention, DR = 0.10 and 0.15	150	3916.91	0.00	2781.01	0.71	0.21	18.06	1.45

DR = dilution rate; sugars* = sum of xylose, glucose, and arabinose; LA = lactic acid. By-products such as ethanol, acetic, formic, and succinic acids were not detected in any of the studied cases.

**Table 2:** Main assumptions considered in the biorefinery simulation.

	Krajnc et al. [33]	Lorenz [36]; Zeist et al. [35]	Dorst [37]; Zeist et al. [35]	This Work
Granulated Sugar (kg product·tonne^−1^ sugar beet)	137	160	167	155
Lime Fertilizer (CaCO_3_) (kg product·tonne^−1^ sugar beet)	51	8	31	30
Molasses (kg product·tonne^−1^ sugar beet)	29	30	39	33
Sugar beet pulp (dry) (kg product·tonne^−1^ sugar beet)	45	60	26	44
Sugar beet pulp (wet) (kg product·tonne^−1^ sugar beet)	-	-	118	198
Beet tops and tails (kg product·tonne^−1^ sugar beet)	11	10	9	10
Beet soil (kg product·tonne^−1^ sugar beet)	98	40	4	47
Sugar content (%)	-	-	-	17.1
Pulp moisture (%)	-	-	-	78.0
Sugar extraction efficiency (%)	-	-	-	98.0
Raw Juice—Water soluble solids concentration (°Brix)	-	-	-	14
Concentrated Juice—WSS concentration (°Brix)	-	-	-	65
Fermentation Yield (*g*/*g*)	-	-	-	0.46
Distillation efficiency (%)	-	-	-	99.0
Dehydration efficiency (%)	-	-	-	98.0
Anhydrous ethanol specification (%)	-	-	-	99.6

**Table 3:** Composition of the streams resulting from the lactic acid downstream process based on bipolar membrane electrodialysis.

Step	Stream	Vol	Residual Sugars	Lactic Acid	Acetic Acid	N _kjel Total_	P _Total_	SO_4_^2−^	Na^+^	Other Anions *	Other Cations **
		(L)	(g/L)	(g/L)	(g/L)	(mg/L)	(mg/L)	(mg/L)	(mg/L)	(mg/L)	(mg/L)
Fermentation Broth from C2, C3, and C4 Fermentations											
**Microfiltration _(fermentation)_**	_permeate_	62.90	9.88	29.84	nd	506	41	998	12,718	4062	1075
**Nanofiltration**	_permeate_	62.90	9.29	25.88	nd	110	10	383	10,361	3852	744
	_retentate_	6.20	12.62	25.63	nd	2545	192	4049	15,116	1244	2363
**Decolorization_1_**		87.90	9.14	19.11	nd	60	15	279	5993	2780	206
**Softening**		96.10	14.01	17.07	nd	55	14	251	7258	2415	99
**Monopolar electrodialysis**	_concentrate_	17.40	0.00	89.86	8.50	125	21	1319	35,414	13,530	503
	_dilute_	87.90	15.76	0.83	0.86	75	7	5	361	24	9
**Bipolar electrodialysis**	_acid_	13.50	0.00	110.30	nd	64	22	1666	2460	16,990	158
	_base_	22.80	1.67	nd	nd	9	<4	nd	27,100	195	393
	_salt_	9.45	0.00	1.05	nd	36	7	nd	299	108	103
**Decolorization_2_**		18.20	0.00	80.15	nd	47	14	1113	1497	11,607	139
**Anion exchanger**		38.50	0.00	20.92	nd	nd	1	5	664	28	9
**Cation exchanger**		48.50	0.00	17.81	nd	nd	7	5	13	13	1
**Vacuum distillation**	_concentrate_	1.10	0.00	788.80	13.15	126	-	308	697	665	10
	_condensate_	47.10	0.00	nd	2.83	nd	-	0	0	1	0
**Fermentation Broth from C6 Fermentation**											
**Microfiltration _(fermentation)_**	_permeate_	88.00	0.00	31.66	nd	509	509	1808	12,819	4522	1329
**Nanofiltration**	_permeate_	88.50	0.00	28.73	nd	141	141	789	10,881	4342	1007
	_retentate_	7.50	0.00	25.33	nd	2975	2975	7672	14,445	1264	3038
**Softening**		99.50	2.38	27.02	nd	75	4	702	10,934	3935	333
**Monopolar electrodialysis**	_concentrate_	22.45	0.00	107.80	9.37	167	27	2208	41,486	17,310	1394
	_dilute_	83.35	3.02	0.85	nd	118	11	11	408	40	33
**Bipolar electrodialysis**	_acid_	20.70	0.00	114.90	9.80	108	23	2891	7714	18,423	357
	_base_	31.70	0.00	0.50	0.64	17	7	32	26,777	253	757
	_salt_	10.05	0.00	0.82	0.56	58	14	16	597	21	43
**Decolorization**	_1_	26.50	0.00	87.00	7.70	41	19	2395	5349	14,195	154
	_2_	40.00	0.00	55.43	5.26	23	24	1534	2725	8978	61
**Anion exchanger_1_**		78.75	0.00	15.73	2.48	17	<4	3	1522	60	33
**Cation exchanger_1_**		83.20	0.00	14.59	nd	4	<3	3	2	58	1
**Anion exchanger_2_**		83.20	0.00	13.10	1.94	nd	6	2	2	25	1
**Vacuum distillation_1_**	_concentrate_	17.60	0.00	56.75	3.30	18	5	7	11	107	4
	_condensate_	46.00	0.00	0.11	0.98	1	<4	1	1	1	2
**Cation exchanger_2_**		20.40	0.00	59.12	4.58	-	-	8	12	97	9
**Anion exchanger_3_**		20.40	0.00	58.71	4.52	-	-	10	13	25	8
**Vacuum distillation_2_**	_concentrate_	1.35	0.00	819.70	40.00	193	12	26	90	328	43
	_condensate_	19.00	0.00	nd	7.30	9	405	1	1	0	1

Nd = not detected; * PO_4_^3−^-P, Cl^−^, NO_2_-N, NO_3_-N; ** K^+^, Mg^2+^, Ca^2+^, NH_4_^+^-N; formic and succinic acids were not detected.
